# Characterization of Coxsackievirus A6 Strains Isolated From Children With Hand, Foot, and Mouth Disease

**DOI:** 10.3389/fcimb.2021.700191

**Published:** 2021-08-16

**Authors:** Hongbo Liu, Ming Zhang, Changzeng Feng, Shanri Cong, Danhan Xu, Hao Sun, Zhaoqing Yang, Shaohui Ma

**Affiliations:** ^1^Institute of Medical Biology, Chinese Academy of Medical Sciences, and Peking Union Medical College, Kunming, China; ^2^Yunnan Key Laboratory of Vaccine Research Development on Severe Infectious Disease, Kunming, China; ^3^Safety Evaluation Center, Sichuan Institute for Drug Control (Sichuan Testing Center of Medical Devices), Chengdu, China

**Keywords:** Coxsackievirus A6, HFMD, KMB17 cell-adapted strain, biological characteristic, vaccine

## Abstract

Coxsackievirus A6 (CVA6) is a key pathogen causing hand, foot and mouth disease (HFMD). However, there are currently no specific antiviral drugs or vaccines for treating infections caused by CVA6. In this study, human rhabdomyosarcoma (RD), African green monkey kidney (Vero), and human embryonic lung diploid fibroblast (KMB17) cells were used to isolate CVA6 from 327 anal swab and fecal samples obtained during HFMD monitoring between 2009 and 2017. The VP1 genes of the isolates were sequenced and genotyped, and the biological characteristics of the representative CVA6 strains were analyzed. A total of 37 CVA6 strains of the D3 gene subtypes were isolated from RD cells, all of which belonged to the epidemic strains in mainland China. Using the adaptive culture method, 10 KMB17 cell-adapted strains were obtained; however, no Vero cell-adapted strains were acquired. Among the KMB17 cell-adapted strains, only KYN-A1205 caused disease or partial death in suckling mice, and its virulence was stronger than its RD cell-adapted strain. The pathogenic KYN-A1205 strain caused strong tropism to the muscle tissue and led to pathological changes, including muscle necrosis and nuclear fragmentation in the forelimb and hindlimb. Sequence analysis demonstrated that the KYN-A1205 strain exhibited multiple amino acid mutations after KMB17 cell adaptation. Moreover, it showed strong pathogenicity, good immunogenicity and genetic stability, and could be used as an experimental CVA6 vaccine candidate.

## Introduction

Hand, foot and mouth disease (HFMD) is an infectious childhood illness predominantly caused by enteroviruses (EVs) (Solomon et al., 2010; Weng et al., 2017; Fujimoto, 2018; Zhang H. et al., 2019). EVs have been divided into 15 species, namely EV-A – L and rhinovirus A – C, comprising more than 100 serotypes [http://www.picornaviridae.com]. Notably, EV-A71 and Coxsackievirus A16 (CVA16) are recognized as the key pathogens causing HFMD. The former is almost exclusively associated with severe disease (Xing et al., 2014; Liu et al., 2015). However, since 2008, Coxsackievirus A6 (CVA6), belonging to the EV-A group, has gradually become one of the main viruses causing HFMD outbreaks in Europe, the Americas, and Asia (Lott et al., 2013; Montes et al., 2013; Hayman et al., 2014; Ang et al., 2015; Bian et al., 2015; Mirand et al., 2016; Anh et al., 2018). Since 2013, the HFMD etiology monitoring system in many cities in China identified that the CVA6 positivity rate suddenly increased, demonstrating that the virus is one of the key pathogens causing HFMD (Yang et al., 2014; He et al., 2018; Zeng et al., 2018). It has been determined that CVA6 is associated with serious diseases, such as aseptic meningitis, encephalitis and acute flaccid paralysis (Fujimoto, 2018). Li et al. (2018) found that CVA6 accounted for 15.2% and 16.9% of severe HFMD cases in China in 2013 and 2015, respectively. Since then, CVA6 has spread throughout mainland China, leading to serious public concern.

CVA6 strains are segregated into 4 genotypes designated as A, B, C, and D and further subdivided into B1–B2, C1–C2, and D1–D3 sub-genotypes. The Gdula prototype strain isolated in the United States in 1949 is classified as genotype A. Genotype B and C strains circulated in mainland China between 1992 and 2007 and in Shandong province of China in 1996 and India in 2008, respectively. Genotype D circulated from 1999 to 2015 in mainland China, Japan, Finland, Spain, and France. B, C, and D Genotypes are further subdivided into B1-2, C1-2, and D1-3 sub-genotypes, respectively. B1 were detected in 1992 in Shandong, China. B2 circulated from 2004 to 2007 in Guangdong, China; D1 and D2 circulated in Japan in 1999 and in France and Spain from 2008 to 2010 and in Japan and mainland of China from 2006 to 2011, respectively. Since 2008, D3 strains gradually become the predominant strains in Finland, Spain, France, Japan, and China (Song et al., 2017; Hoa-Tran et al., 2020).

Intriguingly, it has also been reported that CVA6 is associated with adult HFMD, which has increased in incidence (Broccolo et al., 2019). It has been identified as an important herpes causing pathogen (Shin et al., 2010; Ramirez-Fort et al., 2014); however, the herpes infection is not reported in China’s disease monitoring and reporting system. Furthermore, the CVA6 infection differs from typical HFMD (Drago et al., 2017; Balestri et al., 2018). Hence, it has been speculated that the disease burden caused by CVA6 might be significantly underestimated. Unfortunately, at present, no antiviral drugs or vaccines targeting CVA6 are available. Thus, the development of a CVA6 vaccine is essential.

Previous studies demonstrated that CVA6 was difficult to propagate in African green monkey kidney (Vero) and human embryo lung diploid fibroblasts (KMB17, MRC-5) cells (Woods and Young, 1988; Prim et al., 2013). The majority of studies utilize human rhabdomyosarcoma (RD) cells to isolate and culture CVA6. Hence, the current research on the CVA6 vaccine primarily focuses on the development of an inactivated vaccine using RD cells as the cell matrix or a virus-like particle (VLP) vaccine, both of which are retained in mouse model (Yang et al., 2016; Zhou et al., 2016). Unfortunately, the existing formalin-inactivated EV-A71 vaccine shows no cross-protection against CVA6 or other EV infections (Zhu et al., 2014; Yi et al., 2017).

In the present study, the biological characteristics, such as genotype, nucleotide and amino acid differences, one-step growth curve, pathogenicity and immunogenicity of different cell-adapted CVA6 strains were preliminarily investigated to guide future research on the CVA6 vaccine.

## Materials and Methods

### Virus Isolation and Adaptive Culture

**Figure 1:**
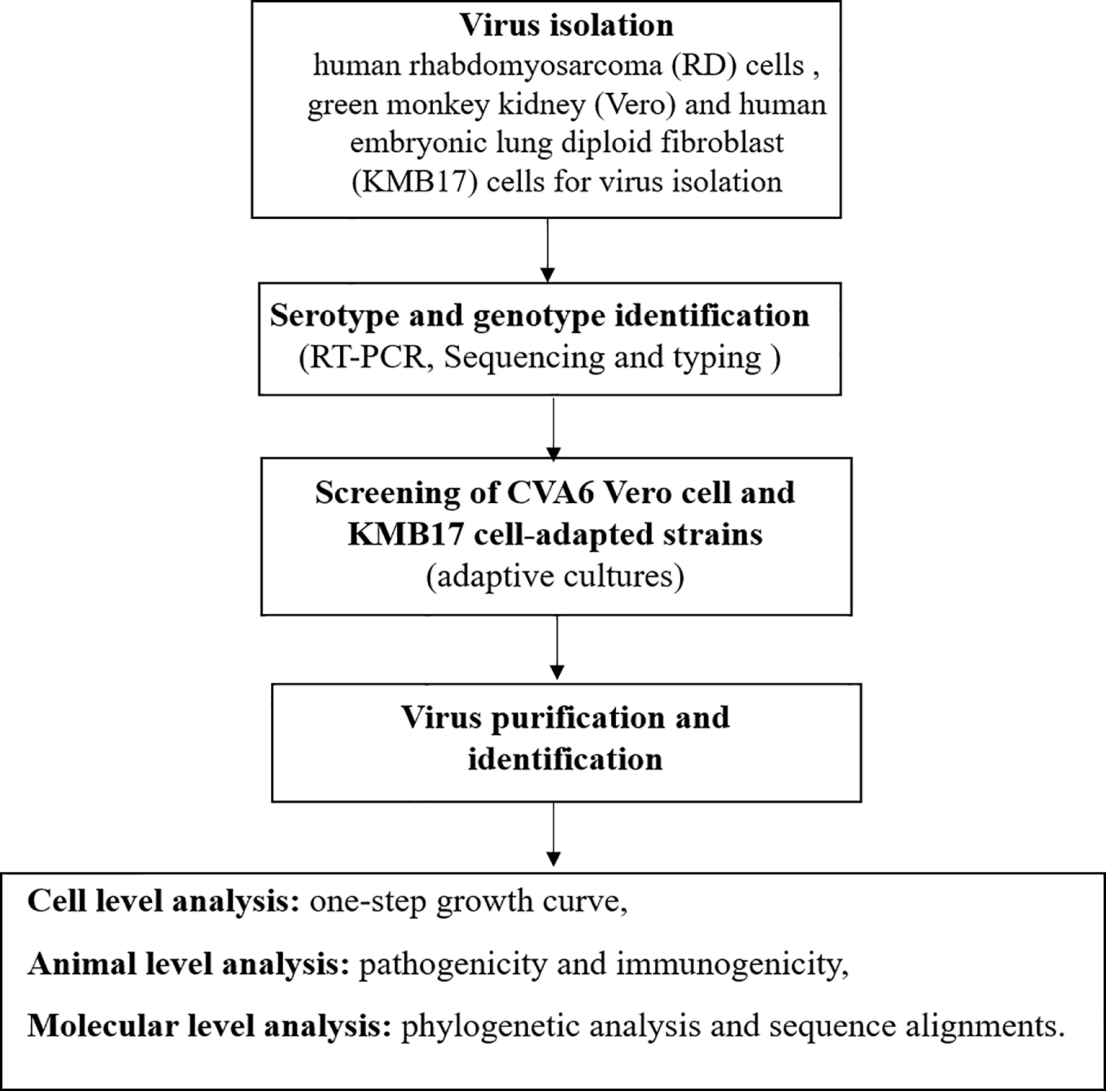
The flow chart of the study.

The stool sample collected from the child was treated using the standard procedure, and adaptively cultured in human rhabdomyosarcoma (RD) cells, African green monkey kidney (Vero) and human embryonic lung diploid fibroblast (KMB17) cells for virus isolation (Prim et al., 2013; Xu and Zhang, 2016). When the cells grew into a dense monolayer, the cell monolayer was washed with PBS three times and the filtered and sterilized samples were inoculated on the cell surface. After adsorption for 2 h, an appropriate amount of maintenance solution and mycoplasma removal agent was added, and the cells were moved to 37°C incubator containing 5% CO_2_ for continuous culture for 7 days. Samples of inducing cytopathic effects (CPE) were considered as positive and were stored at -20°C. After three cycles of freeze-thaw, the next round of passage was carried out. This blind passage was performed for three generations. All of the above operations were performed in a BSL-2 biosafety cabinet. The flow chart of the study is provided in **Figure 1**.

### Reverse Transcription Polymerase Chain Reaction (RT-PCR), Sequencing, and Typing

RT-PCR, sequencing and typing were performed according to a previously described procedure (Liu et al., 2020a). Briefly, the QIAamp Viral RNA Mini Kit (Qiagen, USA) was used to extract the viral RNA from infected cell supernatants. The PrimeScript One Step RT-PCR Kit Ver. 2 (Takara, Dalian, China) was employed to perform RT-PCR. Primers 224 and 222 were used to amplify the partial VP1 sequences. Complete genome fragments were amplified and sequenced using multiple pairs of primers, and the amplification and sequencing primers are summarized in **Table S1**. The positive amplification products were sequenced by Kunming Qingke Biological Technology Co., Ltd. (Kunming, China). The Enterovirus Genotyping Tool was used for EV classification. The VP1-encoding sequences and complete genomes were compared with sequences available in GenBank using the Basic Local Alignment Search Tool (BLAST) [http://www.ncbi.nlm.nih.gov/BLAST]. The VP1 sequences of 37 CVA6 strains characterized in this study were deposited in the GenBank database (accession numbers: MN514784–MN514822).

### Phylogenetic Analysis and Sequence Alignments

The Molecular Evolutionary Genetic Analysis (MEGA) version 7.0 software with the maximum likelihood (ML) method with 1000 bootstrap replications was used to perform the phylogenetic analysis of 127 CVA6 strains (i.e., 90 strains from the GenBank database and 37 strains isolated in this work) and CVA10 prototype strain CVA10/Kowali/AF081300 based on the complete VP1 sequence (915 nucleotides) (Fu et al., 2020). CVA6 was genotyped according to previous studies (Song et al., 2017). CVA6 lineage was defined as forming a monophyletic clade and according to a difference of approximately 15% in the complete VP1 nucleotide sequences was assigned for different genotypes. However, genotypes can be further subdivided into different sub-genotypes, with a difference of over 8% in VP1 nucleotide sequences (Brown et al., 1999). The Geneious 9.0.2 software was employed for pairwise alignment of these sequences.

### Virus Titration

The virus titres were measured using a microtissue culture technique (MCT) according to a previously described protocol (Zhang et al., 2019). Briefly, serially diluted virus samples were inoculated into RD cells in 96-well plates, which were incubated at 37°C in the presence of 5% CO_2_ for 7 days. Subsequently, the 50% cell culture infectious dose (CCID_50_) values were calculated using the Karber method.

### Virus Purification

The CVA6 strains were purified by plaque cloning in RD and KMB17 cells. Briefly, CVA6 strain diluted using a 10-fold gradient (10^−1^–10^−5^) was added to the cell monolayer and incubated at 37°C with 5% CO_2_ for 2 h. Subsequently, the medium was removed and the cell surface was rinsed three times with phosphate-buffered saline (PBS). The cell surface was then covered with 2 mL of MEM containing 0.9% agarose. Following solidification, the inverted culture was incubated at 37°C with 5% CO_2_. When a single plaque was observed under a microscope, it was absorbed with a pipette and dissolved in 500 μL of PBS. The mixture was frozen three times at -20°C and centrifuged at 5,000 × *g* for 30 min. The supernatant was used to inoculate cells in 24-well plates. When the cells demonstrated CPE, the viral RNA was extracted and identified according to the method described above. The virus identified as CVA6 was subjected to next-generation plaque purification. Each strain was purified three times using the same approach.

### One-Step Growth Curve of CVA6 Isolates in RD and KMB17 Cells

Ten CVA6 RD cell-adapted strains and KMB17 cell-adapted strains were inoculated into RD and KMB17 cells with MOI = 1. Three replicates were performed at each time point. After inoculation, the virus solution was harvested every 24 h for 8 days. The virus titres were determined according to the method described above. The one-step growth curve of the virus was plotted as the sample collection time (abscissa) *vs.* virus infection titres (ordinate).

### Experimental Animal Infections

Seven CVA6 RD cell-adapted strains with highly infectious titres and their KMB17 cell-adapted strains were selected and inoculated into the cranial cavity of one-day-old BALB/c suckling mice at a dose of 6.5 lgCCID_50_, and the mice were monitored for 15 days. Specific pathogen-free BALB/c suckling mice were purchased from Hunan Slack Jingda Experimental Animal Co., Ltd., Hunan, China. The experimental mice were infected with the viral stock supernatant by intracranial injection, while the negative control mice were mock infected with uninfected cell supernatant *via* the same route (30 μL/per mouse, 6–10 mice in each group). The pathogenicity of the strain was evaluated based on the clinical characteristics of neonatal mice. The pathogenic strain was selected and inoculated into the cranial cavity of the neonatal mice at 6.5, 5.5, and 4.5 lgCCID_50_. The pathogenicity of the strain before and after KMB17 cell adaptation was compared based on the average body weight, clinical score, survival rate, hematoxylin and eosin (HE) staining, immunohistochemistry results and viral load in each tissue.

### Histopathological and Immunohistochemistry (IHC) Analyses

The histopathological and immunohistochemistry (IHC) analyses were performed according to previously reported methods. Briefly, the tissues of the neonatal mice were dissected and placed in 4% formalin for a week. Following fixation, the tissue sections were embedded in paraffin and stained with HE. After dehydration, the tissue samples were mounted with neutral gum. For IHC analysis, mouse anti-CVA6 antibody (1:1,000 dilution) was incubated at 4°C overnight. Peroxidase-conjugated secondary antibody was added for 50 min at room temperature and then developed using the diaminobenzidine tetrahydrochloride developer solution. All sections were examined under a microscope slide scanner (3D HISTECH Pannoramic 250, Hungary). The acquired images were collected and analyzed.

### Tissue Sampling and RT-qPCR

The heart, liver, spleen, lung, kidney, small intestine and brain tissues of the suckling mice were weighed and ground with a high speed grinder (KZ-II, Servicebio, Wuhan, China). The total RNA was extracted using the TRIzol reagent (Invitrogen, USA). Subsequently, real-time PCR was performed utilizing the One Step PrimeScript™ RT-PCR Kit (Takara) according to the manufacturer’s protocol. Primers CVA6-qP-F (5’-TACCACCGGGARAAACGTCCACG-3’) and CVA6-qP-R (5’-CGGTCAGYTGCAGTGTTAGT-3’) as well as CVA6-probe (FAM-ACGTGAGAGCTTGGGTACMTAGACCCCTTC-BHQ) were used. A sequence of 1468 bp including VP1 fragments amplified by RT-PCR with the primers CVA6-VP1-F (5’-TAATACGACTCACTATAGGGGGTCAGATCTGCAGGTATTAC-3’) and CVA6-VP1-R (5’-GAGGACACCAGAAGATCTCG-3’) was inserted into the pMD18-T plasmid. The recombinant plasmid was further linearized. VP1 RNA obtained from the *in vitro* transcription was then used to evaluate the copies of CVA6.

### Neutralization Assays

The neutralization assays were conducted according to previously described methods. The RD cells (1 × 10^5^/mL) were seeded in 96-well microplates. The serum was serially diluted two-fold by mixing with equal volumes of the CVA6 strains (100 CCID_50_) at 37°C for 1 h. The RD cells were inoculated with the virus-serum mixtures and incubated at 37°C for 7 days.

## Results

### Primary Characterisation of the Virus Isolates

**Figure 2:**
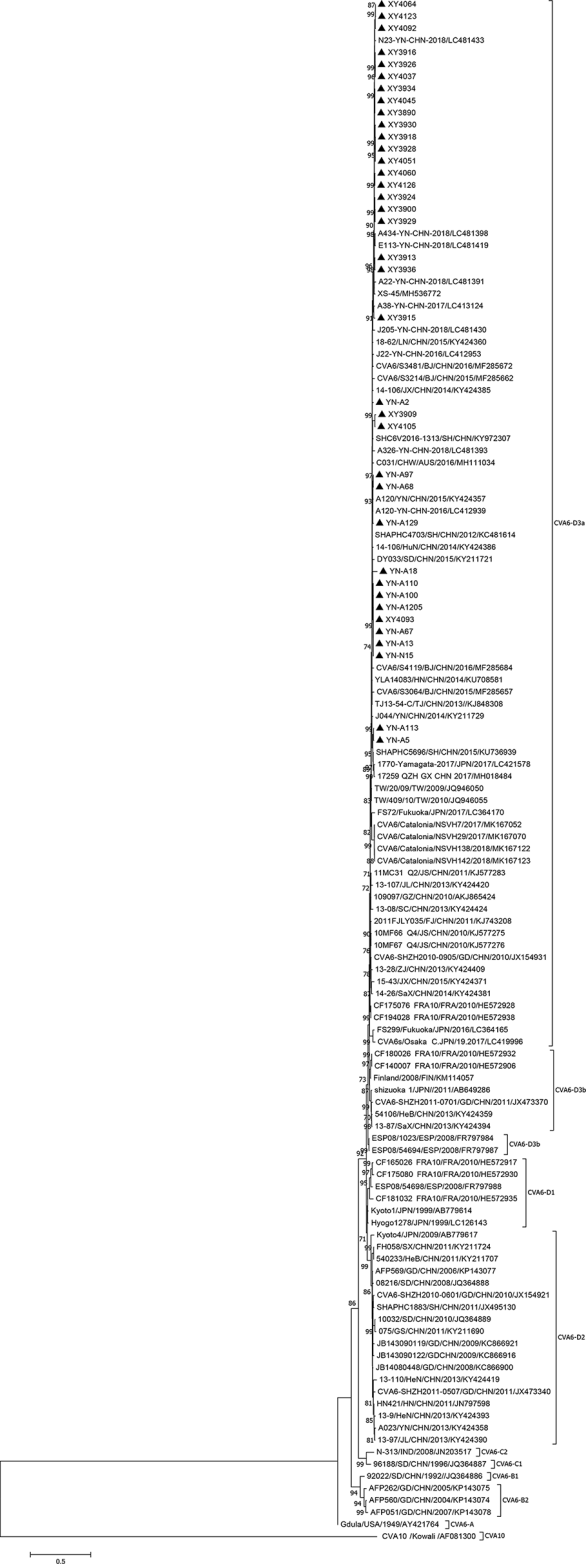
Phylogenetic analysis based on full-length VP1 of CVA6. The 37 CVA6 isolates from this study and 90 representative strains of CVA6 from GenBank and CVA10 prototype strain CVA10/Kowali/AF081300 were used to construct phylogenetic trees based on the full-length VP1 sequence (915 nucleotides) (Test of Phylogeny: Bootstrap method, number of Bootstrap Replications: 1,000 Mode: Kimura 2-parameter model), showing a bootstrap value of > 70%. ▲ indicates the CVA6 strain isolated in this study.

The RD, Vero and KMB17 cells were used to isolate viruses from clinical samples. The virus serotype identification was conducted according to a previously reported approach (Du et al., 2019). A total of 37 CVA6 strains were only isolated from RD cells between 2009 and 2017 during HFMD monitoring in the Yunnan and Hubei provinces, China, but failed to from KMB17 and Vero cells. And by ten adaptive cultures, no CVA6 strain was recovered from Vero and KMB17 cell culture, respectively. 37 CVA6 strains isolated from clinical samples were provided in **Table S2**. The complete VP1 sequences of the 37 strains described in this study had be deposited in the GenBank database under the accession numbers MN514784 to 514822. All isolates belonged to the D3 gene subtype (**Figure 2**). The nucleotide and amino acid homologies of the VP1 sequences were 95.8–100% and 97.0–100%, respectively. After the Vero and KMB17 cells were independently adapted, 10 CVA6 KMB17 cell-adapted strains were obtained and denoted as KYN-A5, KYN-A110, KYN-A1205, KYN-N15, KXY4051, KYN-A13, KYN-A100, KYN-A129, KYN-A68, and KYN-A2. However, no CVA6 Vero cell-adapted strains were acquired. The polyclonal anti-KYN-A1205 antiserum completely neutralized nine CVA6 KMB17 cell-adapted strains (the highest neutralization titre reached 1:1024), indicating that the strains exhibited good immunogenicity.

### One-Step Growth Curve of Different Cell-Adapted Strains

**Figure 3:**
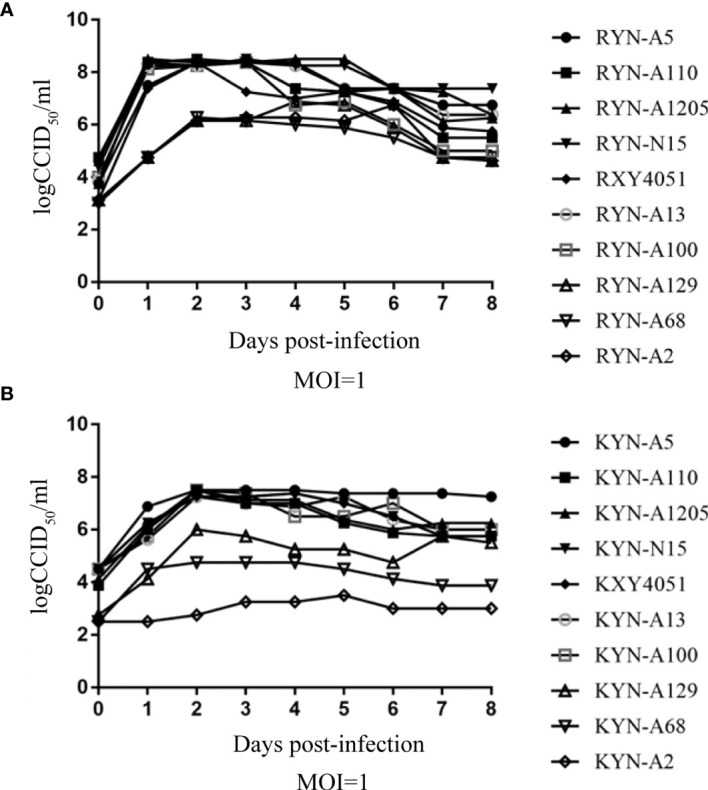
The one-step growth curve of these high-titre RD cell-adapted strains and their KMB17 cell-adapted strains. Panel **(A)** (RD cell lines) and Panel **(B)** (KMB17 cell lines).

The one-step growth curve of these high titre RD cell-adapted strains and their KMB17 cell-adapted strains, were plotted (**Figure 3**). It is noteworthy that at each time point, the virus titres of the YN-A129, YN-A68 and YN-A2 strains in the two studied cell lines were lower than the titres of the other seven strains. By pairwise comparisons of the nucleotide and amino acid sequences of the three whole genomes, no specific mutations were found. Thus, the seven strains with higher titres (> 6.0 logCCID_50_) were used in the subsequent investigations.

### Pathogenicity Analysis

**Figure 4:**
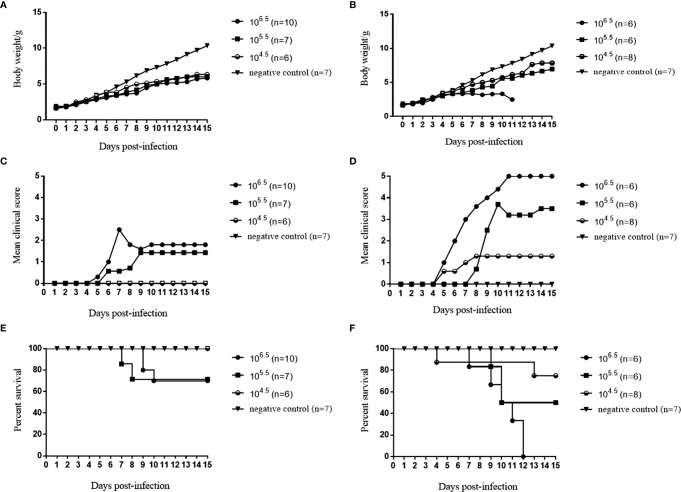
Comparison of virulence of the RYN-A1205 and KYN-A1205 strains. The body weight, mean clinical score and percent survival of neonatal mice injected with the RYN-A1205 strain **(A, C, E)**. The mean clinical score and percent survival of neonatal mice injected with the KYN-A1205 strain **(B, D, F)**. Negative control mice were administered an equal volume of an uninfected cell supernatant instead of the virus.

Among the seven selected CVA6 RD cell-adapted strains, the RYN-A1205, RYN-N15, RYN-A13 and RXY4051 strains were pathogenic to suckling mice, with the RXY4051 strain exhibiting the strongest virulence (**Figure S1**). The course of morbidity was comparable. Decreased vitality, limb weakness and paralysis of the forelimb or hindlimb muscles were observed. Moreover, among the CVA6 KMB17 cell-adapted strains, only KYN-A1205 caused clinical symptoms in suckling mice (**Figure S2**). Hence, the RYN-A1205 strain and its KMB17 cell-adapted strain were inoculated into the cranial cavities of 1-day-old BALB/c suckling mice. The results demonstrated that the RYN-A1205 strain had an increase in virulence after the KMB17-adaption (**Figure 4**).

### Immunohistochemical and Histopathological Analyses

**Figure 5:**
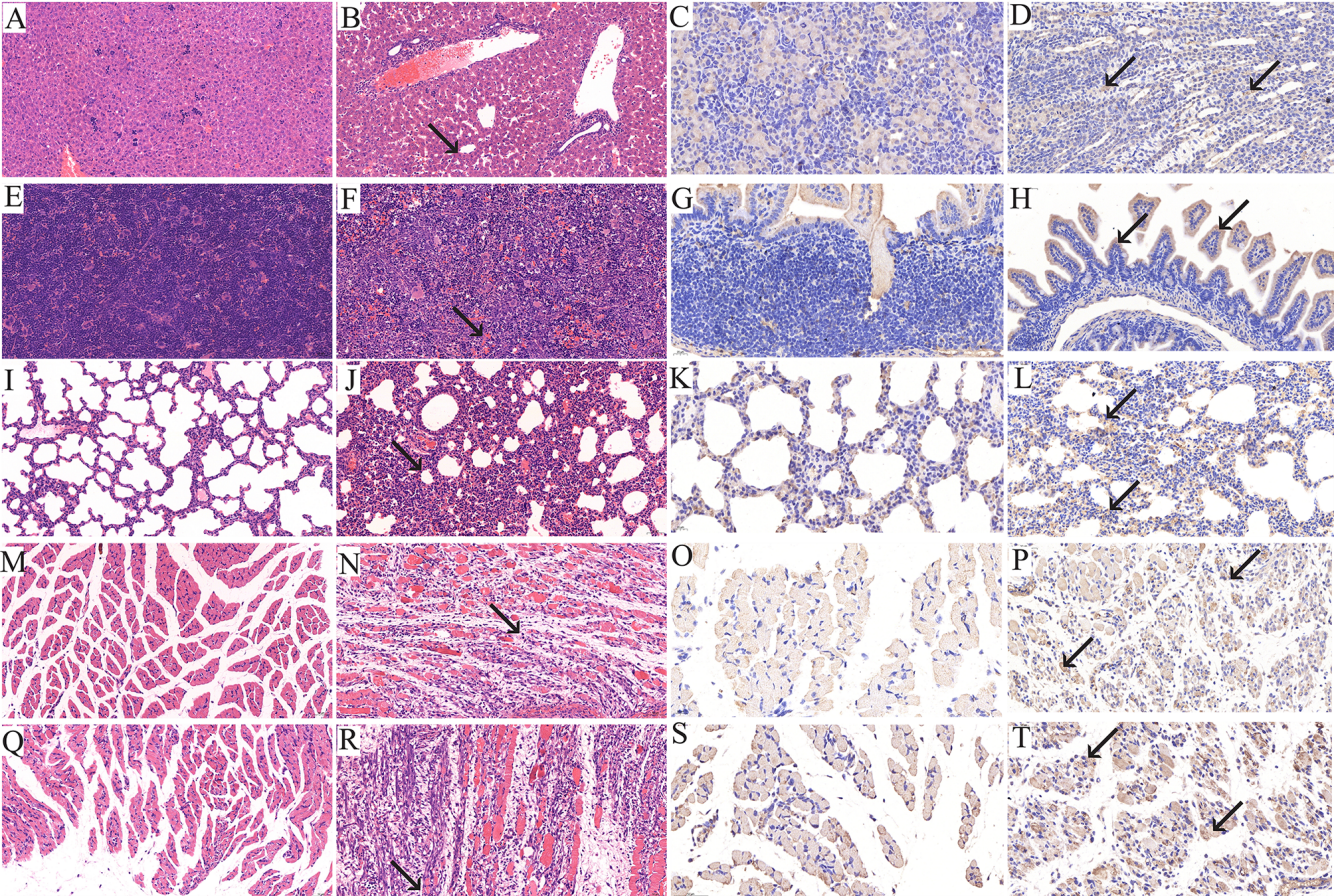
Histopathological (×200) and immunohistopathological analyses of tissues isolated from CVA6-infected neonatal mice (×400). One-day-old BALB/c mice were intracranially injected with 5.5 lgCCID_50_ of the KYN-A1205 strain or an equal volume of uninfected cell supernatant. Obvious histological changes in the liver, spleen, lung, forelimb muscle and hindlimb muscle tissues of the experimental group (**B, F, J, N, R**, black arrow) were noted. No histological changes were observed in the liver, spleen, lung, forelimb muscle and hindlimb muscle tissues of the negative control group **(A, E, I, M, Q)**. The CVA6 antigen was detected in the liver, lung, intestine, forelimb muscle and hindlimb muscle tissues of the experimental group (**D, H, L, P, T**, black arrow). No antigen was detected in the liver, lung, intestine, forelimb muscle and hindlimb muscle tissues of the negative control group **(C, G, K, O, S)**.

When the clinical score reached grades 4–5, the newborn mice were sacrificed and their heart, liver, spleen, lung, kidney, intestine, brain, forelimb muscle and hindlimb muscle tissues were taken for IHC and HE staining. The results revealed that after infection with the RYN-A1205 and KYN-A1205 strains, the number of leukocytes in the liver sinus increased and minor infiltration of neutrophils was noted (**Figure 5**). Moreover, large extramedullary hematopoietic foci in the red pulp and multinucleated giant cells were detected. The alveolar wall was considerably thicker due to infiltration of numerous lymphocytes and neutrophils. The number of large areas of muscle fibres in the forelimb muscle and hindlimb tissues decreased. The muscle fibres appeared necrotic, mitotic or dissolved, which was accompanied by increased lymphocyte and neutrophil infiltration. Additionally, a large number of fibroblasts and some hyperplastic connective tissues were observed. Other tissues showed no significant pathological changes. The kidney, lung, small intestine, forelimb muscle and hindlimb muscle tissues displayed distinct CVA6 antigen distribution, which was evidenced by dark brown staining color. In contrast, the remaining tissues exhibited no antigen distribution.

### Tissue Viral Loads in CVA6-Infected Mice

**Figure 6:**
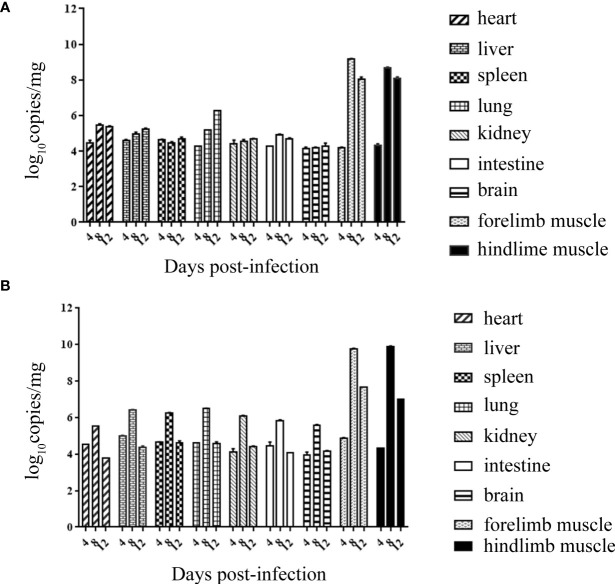
Viral loads of all tissues of suckling mice on the 4th, 8th and 12th dpi with the RYN-A1205 **(A)** and KYN-A1205 **(B)** strains.

Both RYN-A1205 and KYN-A1205 strains were inoculated into the brains of suckling mice at a dose of 5.5 lg CCID_50_. The viral loads of the heart, liver, spleen, lung, kidney, intestine, brain, forelimb muscles, hindlimb muscles of the suckling mice were measured on the 4th, 8th and 12th days after infection. The results showed that there was no significant difference in the viral load of this strain before and after the KMB17 cell adaptation, and all of the suckling mouse tissues showed strong muscle phagocytosis (**Figure 6**). The virus load of the front and hind limbs of the suckling mouse was the highest on the 8th day post infection (dpi) among all tissues.

### Sequence Analysis of Different Cell-Adapted Strains

The complete genome of the RXY4051, KXY4051, RYN-A1205, and KYN-A1205 strains were obtained by Sanger sequencing (accession numbers: MT364500, MT364501, MN184852, MN184852, respectively). The full-length genomes of these four strains are 7351 nt, 7352 nt, 7442 nt, and 7397 nt, the 5’UTR is 696 nt, 697 nt, 748 nt, and 748 nt, and the 3 ‘UTR is 49 nt, 50 nt, 91 nt, and 46 nt, respectively. All contain an open reading frame of 6606 nucleotides, encoding a polyprotein containing 2201 amino acids. P1 structural protein has 870 amino acids, and P2 and P3 non-structural proteins encode 578 and 753 amino acids, respectively. Among them, the complete genome nucleotide and amino acid homology of the RXY4051 and KXY051 strains were 94.2% and 97.9%, respectively (**Table S3**). There are 48 amino acid differences between these two strains (**Table S4**), five amino acid differences in the VP2 region; four amino acid mutations in the VP3 region; and ten amino acid differences in the VP1 region. There are three, three, two, two, eighteen amino acid differences in 2A, 2B, 3A, 3C, and 3D, respectively. In addition, the NCBI BLAST tool was used to compare the strains with the most homologous nucleotide sequences to the respective gene regions of the RXY4051 and KXY4051 strains (**Table S5**). The results showed that the complete genomes of RXY4051 and KXY4051 strains were the most homologous to CVA6/XS45/MH536772 (98.30%) and CVA6/S2792/BJ/CHN/2014/MF285648 (98.35%), respectively, and other gene regions were most homologous to the CVA6 strains, suggesting that the two strains have not recombined with other serotype viruses. In addition, the complete genome nucleotide and amino acid sequence homology of the RYN-A1205 and KYN-A1205 strains were 99.80% and 99.40%, respectively. Compared with the RYN-A1205 strain, a total of 15 nucleotide mutations occurred in the strain KYN-A1205, resulting in 12 amino acid substitutions (**Table S6**).

## Discussion

Following the adoption of the EV-A71 vaccine, the virus spectrum of HFMD has gradually changed. Recently, CVA6 has become the main pathogenic serotype in several regions. CVA6 strains have been segregated into A, B, C, and D, and B1–B2, C1–C2, and D1–D3 subgenotypes. Since 2008, genotype D3 strain gradually becomes the main epidemic strain worldwide. In addition, although sporadic substitutions were found among a few CVA6 epidemic strains, the amino acid sequences were highly conservative by predicted putative functional loops located in the VP4, VP2, VP3, and VP1 of CVA6 (Hoa-Tran et al., 2020). And Loop prediction is consistent with neutralizing linear epitopes from previous report (Xu et al., 2017). Thus, D3 strain is a candidate for developing a CVA6 vaccine (Hoa-Tran et al., 2020).

The CVA6 D3 subtype epidemic strains exhibit stronger transmission ability, infectivity and virulence, which might be associated with a CVA6 epidemic (Song et al., 2017). Based on the nucleotide sequence alignment and phylogenetic analysis of the VP1 sequences, all CVA6 isolates evaluated in the present study were of the D3 subtype, which is consistent with the epidemic strains from mainland China and most international isolates. Hence, it was hypothesized that in-depth research into the known CVA6 isolates would provide crucial guidance for the rational design of effective vaccines against CVA6 infection.

The use of cell lines is necessary for the isolation, proliferation and detection of viruses as well as for the production of viral vaccines. Previous studies demonstrated that proliferation of CVA6 on cell matrices produced by Vero, MRC-5 and other cell lines commonly used during vaccine development was challenging (Woods and Young, 1988; Prim et al., 2013). At present, the majority of studies use RD cells to isolate and culture CVA6, which has hindered the development of effective CVA6 inactivated vaccines. Continuous passage is a traditional adaptive screening method, which has been widely employed to screen various viral adaptive strains (e.g. EV-A71, rabies virus, rotavirus, poliovirus and hepatitis A virus). In this work, 37 CVA6 strains were isolated from RD cells, while, isolation from Vero and KMB17 cells was unsuccessful. Following adaptive culture, only 10 CVA6 KMB17 cell-adapted strains were acquired. This was attributed to the changes in the living environment of the virus after infecting different hosts. Moreover, it is known that CVA6, CVA10, EV-A71 and CVA16 are characterized by high amino acid homology (~67%) (Xu et al., 2017). As for CVA6, it is problematic to adapt to Vero and KMB17 cells, which may be related to the inefficient internal ribosome entry site (IRES) in directing translation, its inability to induce the cellular protein synthesis shutoff, and its deviated codon usage with respect to the cell codon usage. Altogether, this may result in competition for the translational machinery and tRNAs, which additionally may not be well adapted to the virus requirements, contributing to the modest productive growth of CVA6 in cell culture (Chavarria-Miró et al., 2021). In addition, host cell receptors are critical for virus invasion and adherence. Previous studies revealed that scavenger receptor class B, member 2 (SCARB2) and P-selectin glycoprotein ligand-1 (PSGL-1) are the specific receptors for EV-A71 and CVA16, whereas KREMEN1 is a host cell receptor for CVA6 and CVA10 (Nishimura et al., 2009; Yamayoshi et al., 2009; Zhu et al., 2018). The surface loops of VP1 (i.e., BC, DE, EF and HI loops) are the preferred binding sites for numerous small RNA virus receptors. The significant differences in the capsid protein structures in these four loops have been previously reported for CVA6, EV-A71 and CVA16. Hence, the varying arrangement of these four loops could result in many unique capsid surfaces, which might explain why CVA6, EV-A71 and CVA16 do not share the same receptors.

It is recognized that animal models are crucial for vaccine research. Yang et al. found that the RD cell-adapted strain CA6/141 showed a strong tendency towards skeletal muscle and skin (Yang et al., 2016). Large antigen distribution was detected in the skeletal muscles of the hindlimbs and spine. In addition, Zhang et al. reported that the CVA6 RD cell-adapted strain WF057R caused significant pathological changes in the brain, hind skeletal muscles and lungs of infected neonates (Zhang et al., 2017). In the current study, three representative strains exhibiting high pathogenicity caused obvious damage to the forelimb and hindlimb muscles. Furthermore, RYN-A1205 and KYN-A1205 caused damage to the liver, spleen and lungs of the infected mice. These outcomes demonstrated that the CVA6 strain showed strong tropism to the muscles of suckling mice, which might lead to paralysis of the newborn animals and an inability to obtain breast milk, thereby accelerating their death. Other pathological changes might be related to the age of the experimental mice, virus strain, inoculation route and dose. Compared with EV-A71 and CVA16, CVA6 showed weaker tropism to the nervous system and myocardium of suckling mice, which was consistent with the previously reported clinical findings. EV-A71 and CVA16 are typically associated with severe HFMD and nervous system complications; however, severe HFMD caused by CVA6 is rare.

VP1 plays an important role in the virulence and immunogenicity of EVs. The change of the VP1 protein may be related to the symptoms seen in the nervous system caused by EV-A71 infection (Zhang et al., 2014). Similarly, through KMB17 cell adaptation, the 90th, 140th, 240th and 305th amino acids of the VP1 coding region of the RYN-A1205 strain were mutated, and the 305th amino acid substitution was located in the epitope of the CVA6 antigen. The effect of the change at this amino acid position on the immunogenicity of the RYN-A1205 strain requires further examination.

Evaluation of virus strain immunogenicity is critical to vaccine research. The results of the present study revealed that the maximum neutralizing titre of a polyclonal antiserum of the KYN-A1205 strain reached 1:1024, which completely neutralized nine other KMB17 cell-adapted strains. This indicated that KYN-A1205 displayed good and stable immunogenicity. Moreover, in our previous research, KYN-A1205 was passed to the 15th generation in KMB17 cells. The nucleotide and amino acid sequence homology of the P1, P5, P10 and P15 generation subviruses was established at 99.97%–100% and 99.90–100%, respectively, indicating good genetic stability (Liu et al., 2020b). In summary, we identified a CVA6 KMB17 cell-adapted strain KYN-A1205 with good pathogenicity and immunogenicity. KYN-A1205 could be a promising candidate strain for the development of a CVA6 vaccine.

## Data Availability Statement

The datasets presented in this study can be found in online repositories. The names of the repository/repositories and accession number(s) can be found in the article/**Supplementary Material**.

## Ethics Statement 

The studies involving human participants were reviewed and approved by the Human Ethics Commission at the Institute of Medical Biology, Chinese Academy of Medical Sciences. Written informed consent to participate in this study was provided by the participants’ legal guardian/next of kin. The animal study was reviewed and approved by the animal ethics committee, Institute of Medical Biology, Chinese Academy of Medical Sciences (approval number: DWSP201804001).

## Author Contributions

HL, ZY, and SM conceived the study and drafted the paper, HL, MZ, SC, and DX performed the experiments. HS, CF, and ZY helped to interpret results and contributed to the writing. All authors contributed to the article and approved the submitted version.

## Funding

This work was supported by the Research Projects of Yunnan Province, China (Grant Number: 20200AA100009 and 2017FA006), CAMS Innovation Fund for Medical Sciences (CIFMS) (Grant Number: 2016-I2M-1-019 and 2016-I2M-3-026), and Yunnan Leading Medical Scientist Training Program (Grant Number: L-2018003).

## Conflict of Interest

The authors declare that the research was conducted in the absence of any commercial or financial relationships that could be construed as a potential conflict of interest.

## Publisher’s Note

All claims expressed in this article are solely those of the authors and do not necessarily represent those of their affiliated organizations, or those of the publisher, the editors and the reviewers. Any product that may be evaluated in this article, or claim that may be made by its manufacturer, is not guaranteed or endorsed by the publisher.
