# Supplementary material for: Characterization of Coxsackievirus A6 Strains Isolated From Children With Hand, Foot, and Mouth Disease
Source: Front Cell Infect Microbiol. 2021 Aug 16;11:700191. doi: 10.3389/fcimb.2021.700191 (PMC8418080; doi:10.3389/fcimb.2021.700191)
Supplement: Supplementary file 1 [file DataSheet_1.doc]

Supplementary Material

**Table S1** Amplification and sequencing primers of the whole genome sequence.

| Name | Primer sequences (5’→3’) | Position | |
| --- | --- | --- | --- |
| 224 | GCIATGYTIGGIACICAYRT | | 2157-2176 |
| 222 | CICCIGGIGGIAYRWACAT | | 2913-2895 |
| E201F | TTAAAACAGCCTGTGGGTTG | | 1-20 |
| CVA161f | CGTCAGCTAGTAGACAGGAC | | 863-882 |
| CVA62R | GTATCTGGTGGCACTACGA | | 2339--2321 |
| CVA63F | GTTAATGAGGCGAGTGTGG | | 2653-2671 |
| CVA61f | CTGACCGTGCAAGCATAACCAC | | 3126-3127 |
| CVA62f | TTGCCCGATGTGATTGTCA | | 3515-3533 |
| CVA63f | CAACTGCCATTGTTGGAGAACC | | 4225-4226 |
| CVA6-3ff | CAATTAAGAGATAGGAAGTCC | | 4975-4995 |
| CVA6-4f | CCAGCACCAGATGTTATTAGTG | | 5137-5158 |
| CVA6-5f | TTATTGGAATTCACATAGGAGG | | 5885-5906 |
| CVA68R | GCTATTCTGGTTATAACAAATTTA | | 7429-7404 |

**Table S2** Result of clinical sample isolation.

| Clinical number | Sampling time  （year） | Sampling site | Sensitive cells | Clinical symptoms | Gender | Age (year) |
| --- | --- | --- | --- | --- | --- | --- |
| XY3890 | 2017 | Xiangyang | RD | Hand and foot rash, Oral ulce | Male | 2 |
| XY3900 | 2016 | Xiangyang | RD | Hand and foot rash, Oral ulce 1。 | Male | 0.7 |
| XY3909 | 2017 | Xiangyang | RD | Hand and foot rash, Oral ulce | Female | 3.9 |
| XY3913 | 2017 | Xiangyang | RD | Hand and foot rash, Oral ulce | Female | 3 |
| XY3915 | 2016 | Xiangyang | RD | Hand and foot rash, Oral ulce | Female | 1.1 |
| XY3916 | 2017 | Xiangyang | RD | Hand and foot rash, Oral ulce | Male | 1 |
| XY3918 | 2017 | Xiangyang | RD | Hand and foot rash, | Male | 2 |
| XY3924 | 2017 | Xiangyang | RD | Hand and foot rash, Oral ulce | Male | 2.2 |
| XY3926 | 2017 | Xiangyang | RD | Hand and foot rash, Oral ulce | Male | 3.6 |
| XY3928 | 2017 | Xiangyang | RD | Hand and foot rash, | Female | 3.8 |
| XY3929 | 2017 | Xiangyang | RD | Hand and foot rash, Oral ulce | Male | 1.3 |
| XY3930 | 2017 | Xiangyang | RD | Hand and foot rash, Oral ulce | Male | 1.4 |
| XY3934 | 2017 | Xiangyang | RD | Hand and foot rash, | Male | 3.3 |
| XY3936 | 2017 | Xiangyang | RD | Hand and foot rash, Oral ulce | Female | 3.9 |
| XY4037 | 2017 | Xiangyang | RD | Hand and foot rash, Oral ulce | Female | 2.1 |
| XY4045 | 2017 | Xiangyang | RD | Hand and foot rash, Oral ulce | Male | 2.8 |
| XY4051 | 2017 | Xiangyang | RD、KMB17 | Hand and foot rash, | Male | 1.4 |
| XY4060 | 2017 | Xiangyang | RD | Hand and foot rash, Oral ulce | Male | 1.2 |
| XY4064 | 2017 | Xiangyang | RD | Hand and foot rash, Oral ulce | Female | 1.6 |
| XY4092 | 2017 | Xiangyang | RD | Hand and foot rash, Oral ulce | Female | 1.8 |
| XY4093 | 2017 | Xiangyang | RD | Hand and foot rash, | Male | 2.8 |
| XY4105 | 2017 | Xiangyang | RD | Hand and foot rash, Oral ulce | Male | 1.1 |
| XY4123 | 2017 | Xiangyang | RD | Hand and foot rash, Oral ulce | Female | 3.4 |
| XY4126 | 2017 | Xiangyang | RD | Hand and foot rash, Oral ulce | Female | 1.4 |
| YN-A100 | 2015 | Xiangyang | RD、KMB17 | Hand,and foot rash | Male | 7 |
| YN-A110 | 2015 | Kunming | RD、KMB17 | Hand and foot rash, Oral ulce | Female | 1 |
| YN-A113 | 2014 | Kunming | RD | hand foot rash, Oral ulce | Male | 3 |
| YN-A1205 | 2015 | Kunming | RD、KMB17 | hand foot rash, Oral ulce | Female | 4 |
| YN-A129 | 2015 | Kunming | RD、KMB17 | hand foot rash, Oral ulc | Male | 2 |
| YN-A13 | 2014 | Kunming | RD、KMB17 | hand foot rash, Oral ulc | Female | 1 |
| YN-A18 | 2015 | Kunming | RD、KMB17 | hand foot rash, Oral ulc | Male | 3 |
| YN-A2 | 2015 | Kunming | RD、KMB17 | hand foot rash, Oral ulc | Male | 3 |
| YN-A5 | 2015 | Kunming | RD、KMB17 | hand foot rash, Oral ulc | Male | 2 |
| YN-A67 | 2015 | Kunming | RD | Hand and foot rash, Oral ulce | Male | 1 |
| YN-A68 | 2015 | Kunming | RD、KMB17 | Hand and foot rash, Oral ulce | Female | 3 |
| YN-A97 | 2015 | Kunming | RD | Hand and foot rash, Oral ulce | Female | 1 |
| YN-N15 | 2015 | Kunming | RD、KMB17 | Hand and foot rash, Oral ulce | Female | 1.6 |

**Table S3** Nucleotide and amino acid homology of the RXY4051 and KXY4051 strains.

| Genomic region | % Nucleotide identity | % Amino acid identity |
| --- | --- | --- |
| 5’UTR | 96.8 | / |
| VP4 | 96.6 | 100 |
| VP2 | 95.8 | 98.0 |
| VP3 | 95.7 | 98.3 |
| VP1 | 95.8 | 96.7 |
| 2A | 96.2 | 98.0 |
| 2B | 96.6 | 99.0 |
| 2C | 96.4 | 99.4 |
| 3A | 95.0 | 97.7 |
| 3B | 93.9 | 100 |
| 3C | 97.1 | 98.9 |
| 3D | 86.1 | 96.1 |
| 3’UTR | 86.1 | / |
| Complete genome | 94.2 | 97.9 |

**Table S4 Amino acid differences between the RXY4051 and KXY4051 strains.**

| Genomic region | Position in gene region | Position in the complete genome | RXY4051 | KXY4051 |
| --- | --- | --- | --- | --- |
| VP2 | 132 | 201 | Val | Ile |
| VP2 | 138 | 207 | Ala | Pro |
| VP2 | 139 | 208 | Thr | Ala |
| VP2 | 145 | 213 | Lys | Arg |
| VP2 | 151 | 220 | Ala | Thr |
| VP3 | 4 | 329 | Thr | Ala |
| VP3 | 65 | 390 | Ser | Asn |
| VP3 | 180 | 505 | Ile | Thr |
| VP3 | 220 | 545 | Lys | Glu |
| VP1 | 20 | 585 | Thr | Ile |
| VP1 | 98 | 663 | Leu | Pro |
| VP1 | 138 | 702 | Asn | Ser |
| VP1 | 139 | 703 | Asn | Asp |
| VP1 | 174 | 739 | Ile | Val |
| VP1 | 179 | 744 | Ser | Arg |
| VP1 | 235 | 800 | Glu | Ala |
| VP1 | 283 | 848 | Ala | Thr |
| VP1 | 297 | 862 | Val | Ile |
| VP1 | 305 | 870 | Ser | Phe |
| 2A | 3 | 873 | Leu | Phe |
| 2A | 25 | 895 | Arg | His |
| 2A | 68 | 938 | Lys | Arg |
| 2B | 22 | 1042 | Ala | Ser |
| 2C | 306 | 1425 | Val | Gly |
| 2C | 307 | 1426 | Ile | Val |
| 3A | 11 | 1459 | Ser | Thr |
| 3A | 47 | 1495 | Thr | Ile |
| 3C | 148 | 1704 | Glu | Gly |
| 3C | 177 | 1733 | Gly | Ser |
| 3D | 33 | 1772 | Ile | Val |
| 3D | 67 | 1806 | Ile | Leu |
| 3D | 75 | 1814 | Lys | Arg |
| 3D | 89 | 1828 | Asn | Asp |
| 3D | 93 | 1832 | Ser | Thr |
| 3D | 113 | 1852 | His | Gln |
| 3D | 140 | 1879 | Lys | Arg |
| 3D | 165 | 1904 | Val | Ile |
| 3D | 263 | 2202 | Ile | Val |
| 3D | 304 | 2043 | Ile | Val |
| 3D | 308 | 2047 | Ser | Ala |
| 3D | 342 | 2081 | Ser | Leu |
| 3D | 346 | 2085 | Arg | Lys |
| 3D | 435 | 2174 | Ser | Asn |
| 3D | 436 | 2175 | Ala | Ser |
| 3D | 440 | 2179 | Ile | Val |
| 3D | 442 | 2181 | Ile | Val |
| 3D | 444 | 2183 | Lys | Arg |

**Table S5** Strains exhibiting highest homology to the RXY4051 and KXY4051 strains.

|  | RXY4051 | | | | KXY4051 | |
| --- | --- | --- | --- | --- | --- | --- |
| Genomic region | | Strain | | %Nucleotideidentity | Strain | %Nucleotideidentity |
| 5’UTR | | CVA6/17ES4/QD/CHN/2017/MN689954 | | 99.00 | CVA6/S2792/BJ/CHN/2014/MF285648 | 98.85 |
| VP4 | | CVA6/XS-45/MH536772 | | 99.52 | CVA6/SDDY/D58/China/2015/MH086171 | 99.03 |
| VP2 | | CVA6/XS-45/MH536772 | | 98.31 | CVA6/5069/SH/CHN/2013/KJ541158 | 98.18 |
| VP3 | | CVA6/XS-45/MH536772 | | 99.17 | CVA6/S2792/BJ/CHN/2014/MF285648 | 98.06 |
| VP1 | | CVA6/2735_WZH_GX_CHN_2017/MK839026 | | 99.34 | CVA6/P470/2013/China/KP289694 | 98.90 |
| 2A | | CVA6/XS-45/MH536772 | | 98.44 | CVA6/S1956/BJ/CHN/2013/MF285633 | 99.56 |
| 2B | | CVA6/XS-45/MH536772 | | 98.32 | CVA6/P459/2013/China/KP289686 | 100 |
| 2C | | | CVA6/HK480774/2017/MH049775 | 98.48 | CVA6/HK425607/2013/MH049761 | 98.48 |
| 3A | | | CVA6/XS-45/MH536772 | 98.45 | CVA6/C096/CHW/AUS/2016/MH111045 | 98.84 |
| 3B | | | CVA6/17ES4/QD/CHN/2017/MN689954 | 100 | CVA6/SDLY/L120/China/2015/MH086185 | 100 |
| 3C | | | CVA6/17ES4/QD/CHN/2017/MN689954 | 98 | CVA6/S3809/BJ/CHN/2016/MF285678 | 98.91 |
| 3D | | | CVA6/XS-45/MH536772 | 98.05 | CVA6/Weifang/SD/CHN/2014/KX752785 | 98.85 |
| 3’UTR | | | CVA6/XS-45/MH536772 | 94.00 | CVA6/S3992/BJ/CHN/2016/MF285681 | 94.00 |
| P1 | | | CVA6/XS-45/MH536772 | 98.54 | CVA6/S2792/BJ/CHN/2014/MF285648 | 98.20 |
| P2 | | | CVA6/XS-45/MH536772 | 98.27 | CVA6/Weifang/SD/CHN/2014/KX752785 | 98.85 |
| P3 | | | CVA6/XS-45/MH536772 | 98.10 | CVA6/Weifang/SD/CHN/2014/KX752785 | 98.63 |
| Complete genome | | | CVA6/XS-45/MH536772 | 98.30 | CVA6/S2792/BJ/CHN/2014/MF285648 | 98.35 |

**Table S6** Amino acid mutations between the RYN-A1205 and KYN-A1205 strains.

| Gene regions | Position | RYN-A1205 | KYN-A1205 |
| --- | --- | --- | --- |
| VP4 | 8 | Glu | Gln |
| VP4 | 43 | Lys | Arg |
| VP2 | 132 | Ile | Val |
| VP2 | 138 | Pro | Ala |
| VP2 | 139 | Ala | Met |
| VP3 | 4 | Thr | Ala |
| VP3 | 180 | Thr | Ile |
| VP1 | 90 | Glu | Val |
| VP1 | 174 | Val | Val |
| VP1 | 216 | Gln | His |
| VP1 | 283 | Thr | Thr |
| VP1 | 240 | Arg | Lys |
| VP1 | 305 | Phe | Ser |
| 3D | 457 | Lys | Asn |

**
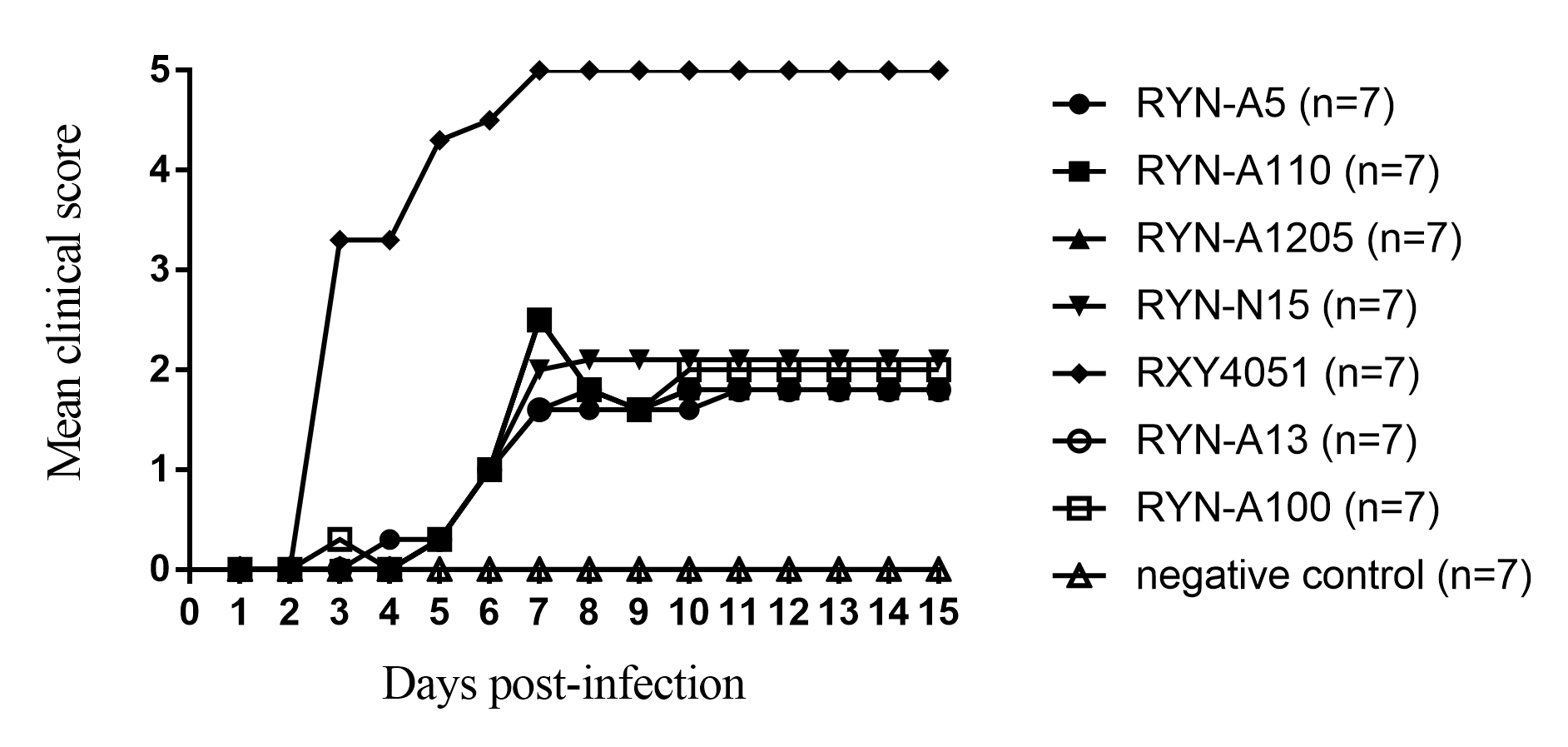
Figure S1** Clinical scores of suckling mice infected with CVA6 RD cell-adapted strains.


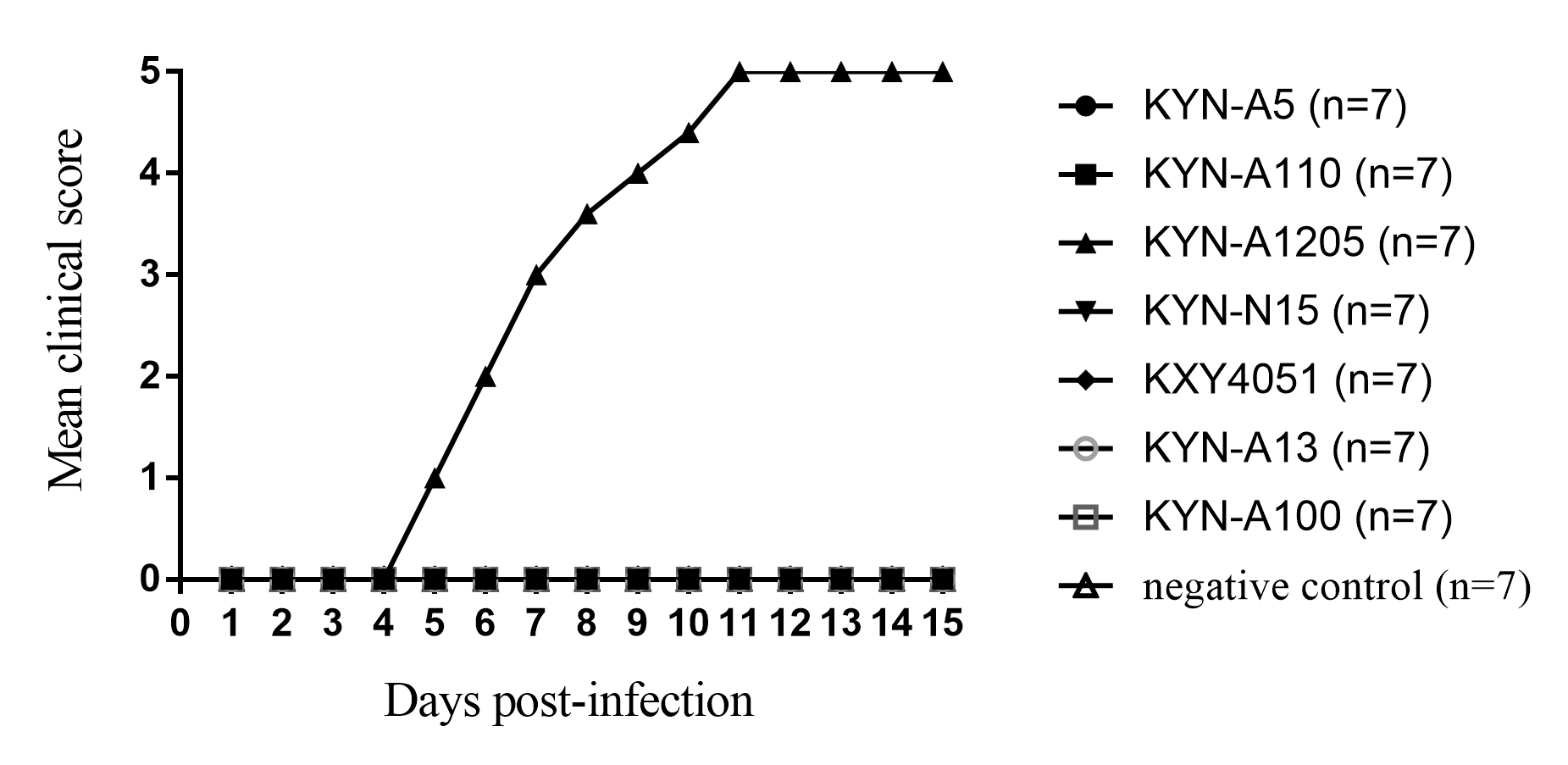


**Figure S2** Clinical scores of suckling mice infected with CVA6 KMB17 cell-adapted strains.
